# Expression of HER-2 in MCF-7 breast cancer cells modulates anti-apoptotic proteins Survivin and Bcl-2 via the extracellular signal-related kinase (ERK) and phosphoinositide-3 kinase (PI3K) signalling pathways

**DOI:** 10.1186/1471-2407-8-129

**Published:** 2008-05-02

**Authors:** Aisha Siddiqa, Linda M Long, Liuxia Li, Robert A Marciniak, Irene Kazhdan

**Affiliations:** 1 Department of Medicine, University of Texas Health Science Center, San Antonio, TX 78229, USA; 2 Department of Cellular and Structural Biology, University of Texas Health Science Center, San Antonio, TX 78229, USA; 3 South Texas Veterans Healthcare Administration, San Antonio, TX 78229, USA

## Abstract

**Background:**

The oncoprotein HER-2 is over-expressed and/or has undergone gene amplification in between 20 to 30% of breast and ovarian cancers. HER-2 amplified breast cancer is associated with a poor prognosis and increased resistance to chemo- and hormonal therapy. Data supporting the transforming potential of HER-2 are irrefutable but the mechanism by which HER-2 contributes to this process is complex and a unified model of HER2-induced increased cell proliferation and survival has not emerged.

To understand the initial event(s) that take place by HER-2 over expression, we studied the effect of short term induction of HER-2 expression in the MCF7 breast cancer cell line.

**Methods:**

We examined the modulation of apoptotic pathways by tetracycline-regulated HER-2 expression for 48 hrs in the MCF7 breast cancer cell line. Specific inhibitors were used to determine signalling pathways that are required for HER-2 induced up-regulation of survivin.

**Results:**

Tetracycline regulated short term over expression of HER-2 in the MCF7 cell line increased the antiapoptotic proteins Bcl-2 and survivin levels. Significant increase of extracellular signal-related kinase (ERK) activation but not AKT1, AKT2 and STAT3 was observed in HER-2 over-expressing MCF7 cells. Specific inhibitors of ERK, and phosphoinositide-3 kinase (PI3K), inhibited the HER-2 induced up-regulation of survivin. We did not observe a change in survivin and NF-κB promoter activity in HER-2 expressing MCF7 cells.

**Conclusion:**

Our results indicate that short term over expression of HER-2 up regulates antiapoptotic proteins Bcl-2 and survivin in MCF7 cells. We determined that survivin is up-regulated via ERK activation and PI3K signalling. Additionally we show that survivin up-regulation is not at transcriptional level. These data provide insight into the mechanism(s) by which induction of HER-2 over expression up-regulates survivin and Bcl-2 and identifies new targets for therapy of breast cancer.

## Background

Impaired apoptosis is critical in cancer development and is a major barrier to effective treatment. Apoptosis is executed by intracellular cysteine proteases called caspases. Two pathways lead to the caspase activation – the extrinsic and intrinsic pathways. The extrinsic pathway is initiated by ligation of death receptors [1]. The intrinsic pathway requires disruption of mitochondrial membranes and release of cytochrome C [2]. Molecules and signalling events that regulate apoptosis affect disease progression and the efficacy of chemotherapy because most chemotherapy kills cancer cells by inducing apoptosis [3].

HER-2 is a key molecule in the regulation of apoptosis in breast cancer cells [4]. Approximately 25–30 percent of breast cancers have amplification of the HER-2/neu gene or over express HER-2, which correlates with poor prognosis and resistance to therapy [5]. The ERBB/HER family of proteins consists of four tyrosine kinase, membrane bound receptors (HER1-4) and more than 13 polypeptide extracellular ligands. HER-2 lacks the capacity to interact with ligand [6], whereas the kinase activity of HER3 is defective [7]. Despite this lack of autonomous ability to respond to a ligand, both HER-2 and HER3 form hetrodimeric complexes with other HER receptors that are capable of generating potent cellular signals. HER-2 containing heterodimers have a higher affinity and a broader specificity for various ligands than the other dimer receptor complexes, owing to slower rates of ligand dissociation. Also, HER-2-containing heterodimers have a slower rate of endocytosis and a higher rate of recycling back to the cell surface. These features translate to potent mitogenic and anti-apoptotic signals [8,9]. HER-2 signalling is mediated by several sequentially activated protein kinases, some of which (ERK, JNK, p38MAPK) belong to super family of mitogen activated protein kinases (MAPK) [10,11]. Other components of the intracellular signalling cascade activated by the ERBB/HER family of receptors include PI3K dependent activation of AKT [12], apoptotic signaling through Bcl-2 [13] and the inhibitor of apoptosis (IAP) families of proteins [14].

The Bcl-2 family plays a pivotal role in the regulation of the mitochondrial "intrinsic" pathway of apoptosis [15]. The Bcl-2 family is subdivided into antiapoptotic members, including Bcl-2 and Bcl-XL, and proapoptotic members, including Bax and Bak [16,17]. Overexpression of antiapoptotic molecules Bcl-2 and Bcl-XL blocks cytochrome C release in response to apoptotic stimuli.

Expression of survivin, an inhibitor-of-apoptosis protein (IAP) family members, is also associated with resistance to apoptosis [18]. Survivin inhibits activation of caspase-9, which is required for the initiation of the intrinsic mitochondrial pathway of apoptosis [19].

In this study we examined the modulation of apoptotic pathways by tetracycline-regulated HER-2 expression in the MCF7 breast cancer cell line. Our results indicate that HER-2 up regulates antiapoptotic proteins Bcl-2 and survivin as reported previously [20,21]. Using specific signalling inhibitors we determined that survivin is up-regulated via ERK activation and PI3K signalling. Additionally we show that survivin upregulation is not at transcriptional level. These data provide insight into the mechanism(s) by which HER-2 over expression protects breast cancer cells from apoptosis and identifies new targets for therapy of breast cancer.

## Methods

### Cell Culture

The human breast cancer cell line MCF-7 was obtained from the American Type Culture Collection (ATCC, Rockville, MD) and maintained in Dulbecco's Modified Eagle's Medium (Invitrogen, Grand Island, NY) supplemented with 10% feotal bovine serum (FBS) (Invitrogen). Cell lines were incubated under standard conditions in a 37°C humidified 5% CO_2 _atmosphere.

### Reagents and Antibodies

FuGENE 6 transfection reagent was obtained from Roche Applied Science (Indianapolis, IN), pTet-On and ptTS plasmids from Clontech (Palo Alto, CA), dual-luciferase reporter assay system from Promega (Madison, CA), ECL™ western blotting detection reagents from Amersham Biosciences (Little Chalfont, Buckinghamshire, UK), BSA protein assay kit from Pierce (Rockford, IL), and U0126, LY294002, AKT inhibitor IV and PD98059 were purchased from Calbiochem (La Jolla, CA).

Donkey anti-rabbit and anti-mouse IgG peroxidase-labeled antibodies and Cy3-conjugated donkey anti-rabbit IgG were obtained from Amersham Biosciences (Piscataway, NJ), rabbit anti-survivin from Novus Biologicals (Littleton, CO), mouse anti-Bcl-2 from (BD Biosciences), rabbit anti-ERB2 from (Delta Bio labs), rabbit ant-pERB2 was from Upstate (Charlottesville, VA), rabbit anti-Akt and rabbit anti-phospho Ribosomal protein S6 (S235) from Cell Signaling Technology (Danvers, MA), rabbit anti-phoso-Akt S473 from (R&D), and rabbit anti-stat3 C-2 was obtained from Santa Cruz Biotechnology (Santa Cruz, CA).

### Inducible expression of HER-2 in the MCF-7 cell line

To produce HER-2 expressing, tetracycline inducible MCF-7 cells, two rounds of stable transfection and selection were used. In the first round, MCF-7 cells were co-transfected with a pTet-On and ptTS at a ratio of 1:10. pTet-ON carries a tetracycline-inducible transcriptional transactivator and ptTS encodes a transcriptional silencer, which binds to the tetracycline response element (TRE) in the absence of tetracycline and decreases background promoter activity. After G418 selection, clones were tested for basal and inducible promoter activity with the tetracycline inducible luciferase reporter plasmid, pTRE-luc (Clontech). The clone that demonstrated the highest ratio of induced/basal activity was then transfected with pTRE-HER2, a construct in which the ErbB2 cDNA was cloned under control of TRE in a pTRE-hygro backbone. After a second round of selection with Hygromycin B, clones were isolated and screened for background and doxycycline-inducible (2 μg/ml) expression of HER-2 by western blotting (described below).

### Western blotting

Cells were lysed in buffer (20 mM Tris-HCl pH 8, 100 mM NaCl, 1 mM EDTA, 1% NP-40). Protein concentrations were determined using the BSA protein assay kit. Twenty-five μgs of whole-cell protein lysate from each sample was diluted in 25 μl of SDS loading buffer (4% SDS, 2% glycerol, 0.01% bromphenol blue, 4% β-mercaptoethanol and 125 mM Tris-HCl, pH 6.8), incubated for 5 min at 98°C and 5 min on ice and then centrifuged at 6000 rpm in a microcentrifuge at room temperature. Proteins in the supernatant were resolved on 10% SDS/polyacrylamide gels and transferred to PVDF membranes. Membranes were blocked for 1 h in 5% nonfat dry milk in TBST (10 mM Tris-HCl pH 7.5, 150 mM NaCl and 0.1% Tween 20) and probed for 2 h with primary antibodies diluted in TBST/5% nonfat dry milk as per the supplier's recommendations. Membranes were then washed with TBST 20 minutes × 3 and incubated for 1 h with horseradish peroxidase-labeled secondary antibodies in TBST. After an additional four washes with TBST, bound antibody was visualized by chemiluminescence.

### Cloning of survivin promoter in pGL3-Basic vector to produce Survivin-Luciferase

Survivin promoter (nucleotides 1821 – 2800, accession U75285) was amplified by PCR from HeLa genomic DNA using primers:

forward 5'-CTGGCCATAGAACCAGAGAAGTGA-3'

reverse 5'-CCACCTCTGCCAACGGGTCCCGCG-3'

[22]. The PCR product was first cloned into T-Vector™(Promega) and subsequently sub-cloned into the pGL3-Basic vector to produce Survivin-Luc. Fidelity of PCR cloning was verified by sequencing survivin promoter. MCF-HER2 cells, untreated or induced with doxycycline for 48 hours, were transiently transfected with Survivin-Luc plasmid

## Results

### Tetracycline-inducible overexpression of HER-2 in MCF7 cell line

**Figure 1:**
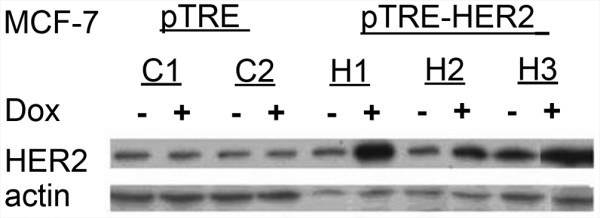
**Doxycycline-inducible expression of HER-2 protein**. Three pTRE-HER2-transfected clones (H1, H2 and H3) and two pTRE-transfected control MCF-7 clones (C1 and C2) were incubated with or without doxycycline. Whole cell protein extracts were collected and evaluated for HER2 expression by western blotting using HER2 antibodies.

The HER-2 cDNA was cloned under the control of tetracycline response element in pTREhygro as described above, to produce the plasmid pTRE-HER2. The MCF7 cell line was transfected with pTRE-HER2, and clones were isolated by selection with hygromycin B and screened for tetracycline inducible expression of HER-2. Doxycyclin-inducible expression of HER-2 was quantified by Western blotting. Three HER-2-transfected clones (H1, H2 and H3) and two vector-transfected control clones (C1 and C2) were incubated with or without doxycycline. Whole cell protein extracts were prepared and evaluated for ErbB2 expression by western blotting (Figure 1). Induction of HER-2 was quantified by NIH Java image processing program ImageJ. In the presence of Dox HER-2 was increased 4.1, 2.7 and 2.8 fold compared to without Dox in H1, H2 and H3 clones respectively. No significant difference in HER-2 with or without Dox was found in the vector control clones (C1 and C2).

### Activated HER-2 up-regulates survivin expression in MCF-7 cells

**Figure 2:**
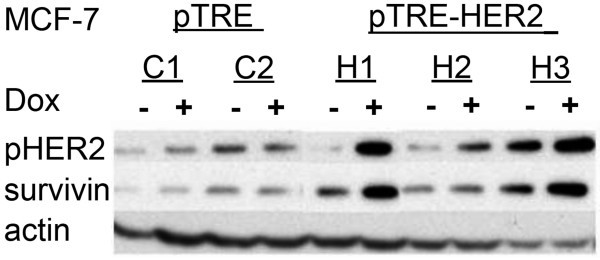
**Activated HER-2 up-regulates survivin expression**. Three pTRE-HER2-transfected (H1, H2 and H3) and two pTRE-transfected control (C1 and C2) MCF-7 clones were incubated with or without doxycycline. Whole cell protein extracts were prepared from untreated cells or cells treated with doxycycline for 48 hours. Extracts (25 ug protein/lane) were analyzed by Western blotting for the expression of survivin and phosphorylated HER-2).

Due to a significant overlap of biological features attributable to the overexpression of HER-2 and survivin (i.e., enhanced proliferation, improved cell survival, resistance to chemotherapy, correlation with poor prognosis, etc) and the report of survivin regulation by HER-2 [21], we investigated survivin regulation in HER-2-overexpressing MCF7 cells. Two control clones (C1 and C2) and three pTRE-HER2 clones (H1, H2 and H3) were grown in the presence or absence of doxycycline for 48 hours. Whole cell protein extracts were prepared and analyzed for expression of survivin and an active, phosphorylated form of HER-2 (phospho-ErbB2). The experiments were repeated twice with separately collected protein extracts. Figure 2 demonstrates pronounced up-regulation of survivin protein in doxycycline-treated cells, which parallels the levels of phospho-HER-2.

### Up regulation of survivin is not due to an increase in G2/M cells in HER 2-overexpressing population

Survivin is preferentially expressed during the G2 and M phases of the cell cycle. We therefore performed a cell cycle analysis to rule out the possibility that up-regulation of survivin is due to merely an increase of a fraction of G2/M cells in the HER-2-overexpressing population. Cells were cultured with (Dox +) or without (Dox -) doxycycline for 48 hours, incubated with propidium iodide and analyzed for cell cycle distribution by flow cytometry. As shown in table 1, no increase in the G2/M cell fraction was found in doxycycline-treated MCF-HER2 cultures.

**Table 1:** Cell cycle distribution of HER2 transfected MCF-7 cells

	G1	S	G2/M
MCF7-HER2 (- Dox)	66.34	14.97	18.68
MCF7-HER2 (+ Dox)	66.68	19.67	13.65

MCF7 Cells were cultured with (+ Dox) or without (- Dox) doxycycline for 48 hours, incubated with propidium iodide and analyzed for cell cycle distribution by flow cytometry.

### Activity of the survivin promoter is not enhanced by HER-2 overexpression in MCF7- cells

**Figure 3:**
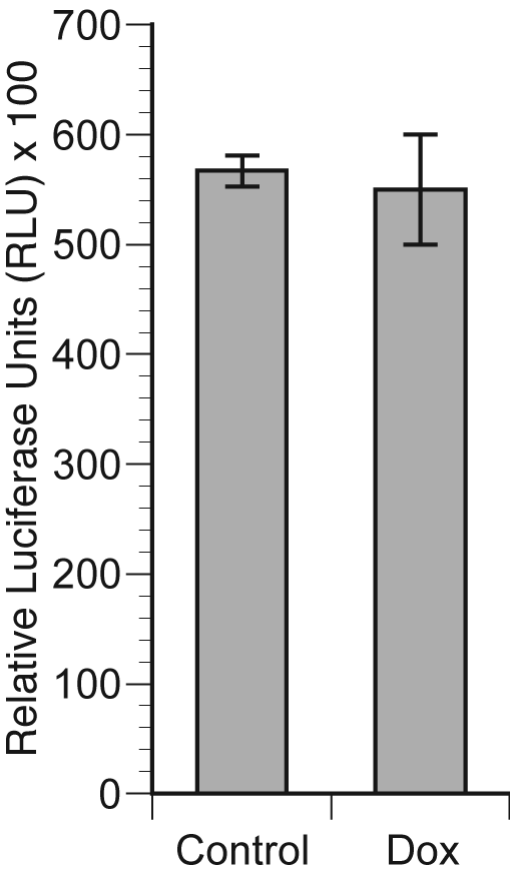
**Activity of the survivin promoter is not enhanced by HER2 over-expression in MCF cells**. MCF-HER2 cells were plated in duplicate wells at a concentration of 5 × 10^4 ^cells per well in 24 well plates. Doxycycline (dox) or saline (control) was added 16 hours later and incubation continued for additional 24 hours, after which cells received fresh serum-containing medium with or without Dox. Cells were then co-transfected with a Survivin-Luc plasmid and pRL-TK, which contains the Renilla luciferase coding sequence under control of the TK promoter as a control for transfection. 48 hours later cells were harvested and dual-luciferase assay was performed per manufacturer's instructions. Data are presented as a ratio of Firefly/Renilla luciferase ×100.

Survivin expression is regulated both at the levels of transcription [22] and protein stability [23]. Understanding the precise level at which ErbB2 up-regulates survivin expression will help to delineate the underlying signal transduction pathways and will allow better targeting of survivin in ErbB2-overexpressing breast cancer cells. We cloned a 976 bp genomic DNA fragment containing the survivin promoter in the reporter plasmid pGL3. MCF7-HER2 cells were plated in duplicate wells at a concentration of 5 × 10^4 ^cells per well in 24 well plates. Doxycycline (dox) or saline control was added 16 hours later and incubation continued for additional 24 hours, after which cells received fresh serum-containing medium with or without Dox. Cells were then co-transfected with Survivin-Luc plasmid and pRL-TK, which contains Renilla luciferase sequence under control of TK promoter. 48 hours later cells were harvested and dual-luciferase assay was performed per manufacturer's instructions. Data are presented as a ratio of Firefly/Renilla luciferase activity × 100 in Figure 3. The activity of survivin promoter was not increased in doxycycline-treated, HER-2-overexpressing cells as compared to cells with background level of HER-2 expression grown in the absence of doxycycline.

### Bcl-2 is up-regulated in HER-2 expressing MCF7 cell line

**Figure 4:**
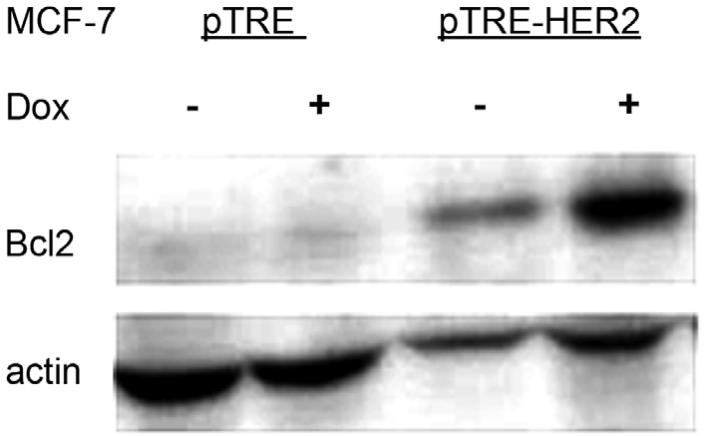
**Bcl-2 is up-regulated in HER-2 expressing MCF7 cell line: **pTRE-HER2 and pTRE-transfected MCF-7 clones were incubated with or without doxycycline for 48 hours. Whole cell protein extracts were collected and analyzed for expression of Bcl-2 by western blotting.

Over expression of Bcl-2 blocks cytochorme C release in response to apoptotic stimuli making cells resistant to many chemotherapeutic agents. We determined the Bcl-2 status in MCF7-HER2 cells. MCF7-HER2 clones were grown in the presence or absence of doxycycline for 48 hours. Whole cell protein extracts were collected and analyzed for expression of Bcl-2 by western blot (Figure 4). Bcl-2 protein is significantly up-regulated in doxycycline-treated MCF7-HER2 cells in which HER-2 expression is induced.

### Activation of the ERK signal transduction pathways in response to HER-2 over-expression

**Figure 5:**
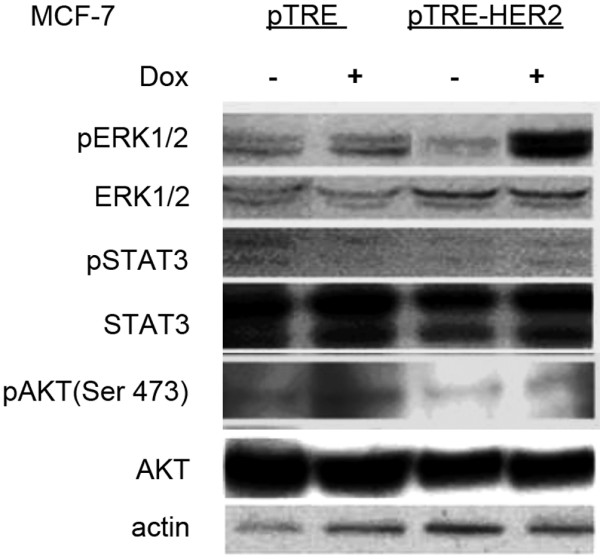
**Analysis of total ERK 1/2, STAT 3, AKT 1 and their phosphorylated forms in HER2-overexpressing and control cells**. pTRE-HER2 and pTRE-transfected MCF-7 clones were incubated with or without doxycycline for 48 hours. Whole cell extracts were prepared and analyzed by Western blotting with the indicated antibodies. Experiments were repeated twice with independently collected extracts.

Over-expression of HER-2 has been reported to activate multiple signal transduction pathways including Ras-MAPK, the PI3-K-Akt-NF-kappaB cascade and STAT 3 [24]. We determined the state of activation of these pathways in MCF7 cells after doxycycline-induced HER-2 overexpression. Vector-transfected (pTRE) cells and MCF7-HER2 cells were incubated with or without doxycycline for 48 hours. Whole cell protein extracts were analyzed by western blotting for expression of total ERK 1/2, STAT 3 and AKT 1 and their respective activated forms (phospho-ERK 1/2, phospho-STAT3 and phospho-AKT-1; Figure 5). There were no changes in the total levels of ERK 1/2, STAT 3 and AKT 1. In accordance with the previous reports, we identified pronounced induction of phospho-ERK 1/2 in doxycycline-treated MCF7-HER2 cells. However, there were no changes in the levels of phospho-STAT 3 and phospho-AKT (Ser473).

### NF-kappaB is not activated in response to induction of HER-2 over-expression in MCF7 cells

**Figure 6:**
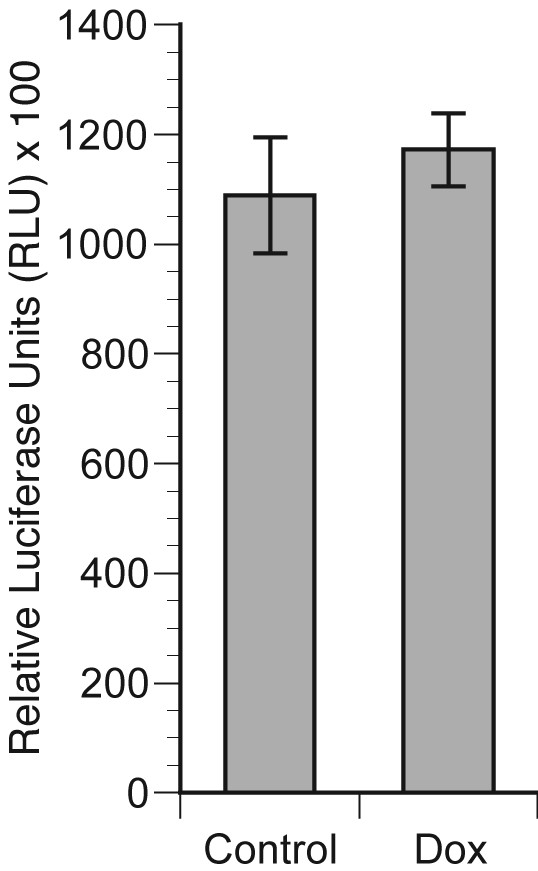
**Activity of NF-kappaB in control and doxycycline-treated MCF7-HER2 cells**. MCF7-HER2 cells were plated in duplicate wells at a concentration of 5 × 10^4 ^cells per well in 24 well plates. Doxycycline (Dox) or saline (control) was added 16 hours later and incubation continued for additional 48 hours, after which cells received fresh serum-containing medium with or without Dox. Cells were then co-transfected with NF-kappaB-RE-Luc plasmid and pRL-TK, which contains Renilla luciferase sequence under control of TK promoter. 48 hours later cells were harvested and dual-luciferase assay was performed per manufacturer's instructions. Data are presented as the ratio of Firefly/Renilla luciferase actvity × 100.

Stimulation of a wide variety of cell-surface receptors leads directly to NF-κB activation and a rapid change in gene expression. To determine whether NF-kappaB is activated in response to induction of HER-2 in MCF7 cells we transiently transfected control and doxycycline-treated MCF7-HER2 cells with a plasmid containing firefly luciferase sequence driven by five tandem consensus NF-kappaB elements. As demonstrated in Figure 6, there was no significant increase in NF-kappaB-mediated luciferase activity in cells with induced HER-2 overexpression as compared to their uninduced counterparts.

### Up-regulation of survivin in HER-2 induced MCF7 cells is via ERK activation and PI3K signalling

**Figure 7:**
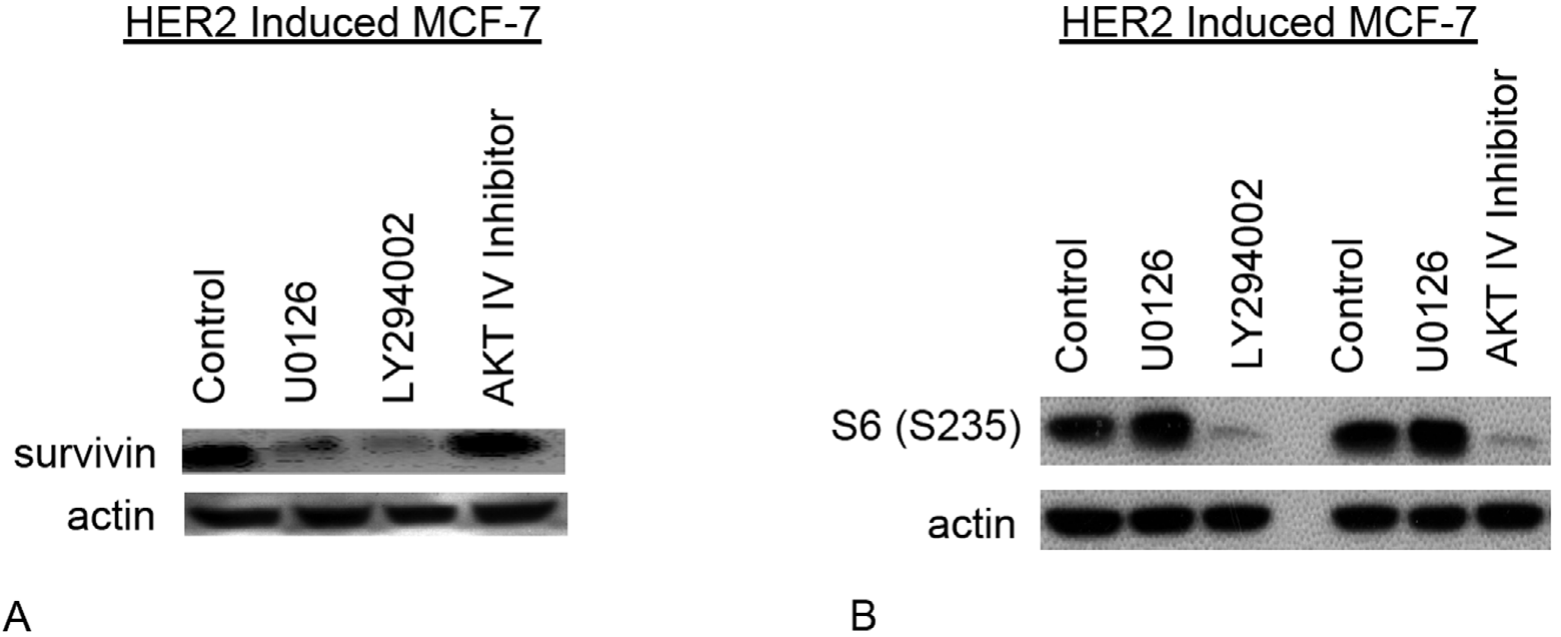
**Signaling pathways involved in survivin regulation in HER2 over expressing MCF7cells**. A- HER2 induced MCF7 cells were incubated for 48 hours with specific inhibitors of PI3K (10 μM LY294002) AKT1/2/3 (15 μM AKT IV Inhibitor) and ERK1/2 (10 μM U0126) or without inhibitor (control) and analyzed by western blotting for expression of survivin. B- To confirm the inhibition of AKT and PI3K, HER2 induced MCF7 cells were incubated for 48 hours with specific inhibitors of PI3K (10 μM LY294002) AKT1/2/3 (15 μM AKT IV Inhibitor). ERK1/2 (10 μM U0126) and without inhibitor MCF-7 cells were used as controls, and analyzed by western blotting for expression of phospho-ribosomal protein S6 (S235).

To investigate the regulation of survivin expression in HER-2 induced MCF7 cells we used specific inhibitors of the signal transduction pathways. HER-2 inducible MCF7 cells were incubated for 48 hours after addition of doxycycline and/or signal pathway inhibitors and analyzed, by western blotting for expression of survivin. We used PI3 kinase inhibitor – LY294002 (10 μM) and AKT IV inhibitor (15 μM) that selectively inhibits the cellular phosphorylation/activation of AKT1/2/3 but does not inhibit known upstream activators of AKT, i.e. PI3K or PDK. To inhibit the MAPK pathway we used the Erk 1/2 inhibitor U0126 (10 μM). As shown in figure 7A, a decrease in survivin up-regulation was observed in the MCF7-HER2 cells treated with ERK 1/2 inhibitor U0126 and PI3 kinase inhibitor – LY294002, suggesting the involvement of these pathways in the up-regulation of survivin. However no significant change was observed by inhibition of activated AKT1/2/3 by AKT IV inhibitor. Inhibition of PI3 kinase and AKT was confirmed by decrease in phosphor-ribosomal protein S6 (S235), the down stream target of phospho-AKT [25] (figure 7B).

## Discussion

Her-2/neu overexpression is associated with poor overall survival and drug resistance in breast and ovarian cancer patients. Although the molecular mechanisms by which Her-2/neu induces drug resistance are not well established, there is increasing evidence that this resistance is a consequence of deregulation of apoptotic pathways in cells. Chemotherapy, radiation, and immunotherapy all rely heavily on apoptosis to kill breast cancer cells [26]. Therefore we investigated the pro- and anti apoptotic regulators and the signal transduction pathway(s) influenced by tetracycline inducible HER-2 over-expression in MCF-7 breast cancer cell line. The advantage of tetracycline inducible HER-2 expression is that the short-term effect of increased HER-2 expression can be determined. We show that acute over expression of HER-2 in MCF7 cells up-regulates the anti apoptotic protein Bcl-2. Bcl-2 is expressed in 70% of breast cancers and it functions directly in apoptosis regulation and in oncogenesis. Oncogenes such as c-Myc, which drive cells to enter the cell cycle, also engage Bax/Bak-dependent apoptosis [27]. Antiapoptotic Bcl-2 family members can prevent such cell death and thereby promote oncogenic transformation. Additionally high expression level of anti-apoptotic Bcl-2 proteins confers a clinically important chemoresistant phenotype on cancer cells[28]. Likewise, reduced expression of pro-apoptotic Bax levels has been associated with poor response to chemotherapy and shorter overall survival for patients with breast cancers, whereas enhanced expression of Bax protein correlate with a good response to chemotherapy in vivo [29].

In the absence of new protein synthesis, survivin protein level is expected to decrease steadily with a half-life of approximately 30 minutes [23]. MCF7-HER2 cells, treated with an inhibitor of protein synthesis cycloheximide show a slower decrease in survivin protein level in HER-2 over-expressing MCF7 cells than in control cells (data not shown), which suggests that up-regulation of survivin is post translational. Expression of survivin in tumours correlates with a poor clinical outcome in a variety of malignancies including breast cancer by providing protection against apoptotic stimuli [30]. It is noteworthy that we did not observe a significant anti- apoptotic effect of survivin up regulation after treatment of HER2 over-expressing MCF7 cells with taxol, adriamycin and herceptin (data not shown). This suggests that unlike stable over expression of HER2, an inducible short term HER2 expression and upregulation of survivin is not enough to exert an anti-apoptotic effect.

Growth and survival signals elicited by activated HER-2 are largely mediated via PI3K-Akt and Ras-MAPK signaling pathways. Using specific PI3K, ERK and AKT inhibitors, we show that PI3K and ERK signalling are responsible for up-regulation of survivin in MCF7-Her2 cells. Inhibition of pAKT did not down-regulate survivin in these cells. The exact role of Akt in this process remains to be determined. Down-regulation of survivin may occur independent of p-Akt expression. PI3K-dependent, but Akt-independent, mechanisms by which HER-2 might regulate survivin include effects on serum- and glucocorticoid-induced kinases (SGK), which are serine/threonine kinases that are highly homologous to Akt and, like Akt, are regulated by PI3K. Although the effects of SGK on survivin have not been studied, SGK regulates cell survival [31] and may therefore be a candidate for regulating survivin protein expression. In addition, PI3K affects cell survival through a protein kinase C-dependent pathway that is mediated by phospholipase Cγ activity [32]. Thus, it is possible that HER-2 regulates survivin in part through PI3K-dependent effects on SGK and/or phospholipase Cγ.

## Conclusion

After a short term (48 hours) induction of HER2 in MCF7 breast cancer cells anti-apoptotic proteins survivin and Bcl-2 are up-regulated. PI3K and ERK but not the NF-kappaB, STAT3 and AKT signalling are involved in up-regulation of survivin. Regulation of survivin in HER-2-positive MCF7 cells is not at transcriptional level. Understanding the regulation of HER-2 signalling pathways will help to identify new targets/strategies for the treatment of patients with tumours that are dependent on HER-2 induced signalling pathways for their survival.

## Competing interests

The authors declare that they have no competing interests.

## Authors' contributions

LML and LL analysed the MCF7- HER2 clones. IK, with the assistance of RAM, designed the study. AS drafted the manuscript and RAM helped in writing the manuscript. All authors read and approved the final manuscript.

## Pre-publication history

The pre-publication history for this paper can be accessed here:


